# Evaluation of Remotely Sensed and Interpolated Environmental Datasets for Vector-Borne Disease Monitoring Using In Situ Observations over the Amhara Region, Ethiopia

**DOI:** 10.3390/s20051316

**Published:** 2020-02-28

**Authors:** Woubet G. Alemu, Michael C. Wimberly

**Affiliations:** 1 Department of Earth and Environment, Florida International University, Miami, FL 33199, USA; 2 Department of Geography and Environmental Sustainability, University of Oklahoma, Norman, OK 73019, USA; mcwimberly@ou.edu

**Keywords:** accuracy assessment, environmental data, EPIDEMIA, AMSR-E, AMSR2, FLDAS, MODIS, TRMM/GPM, CHIRPS, epidemiological data

## Abstract

Despite the sparse distribution of meteorological stations and issues with missing data, vector-borne disease studies in Ethiopia have been commonly conducted based on the relationships between these diseases and ground-based in situ measurements of climate variation. High temporal and spatial resolution satellite-based remote-sensing data is a potential alternative to address this problem. In this study, we evaluated the accuracy of daily gridded temperature and rainfall datasets obtained from satellite remote sensing or spatial interpolation of ground-based observations in relation to data from 22 meteorological stations in Amhara Region, Ethiopia, for 2003–2016. Famine Early Warning Systems Network (FEWS-Net) Land Data Assimilation System (FLDAS) interpolated temperature showed the lowest bias (mean error (ME) ≈ 1–3 °C), and error (mean absolute error (MAE) ≈ 1–3 °C), and the highest correlation with day-to-day variability of station temperature (COR ≈ 0.7–0.8). In contrast, temperature retrievals from the blended Advanced Microwave Scanning Radiometer on Earth Observing Satellite (AMSR-E) and Advanced Microwave Scanning Radiometer 2 (AMSR2) passive microwave and Moderate-resolution Imaging Spectroradiometer (MODIS) land-surface temperature data had higher bias and error. Climate Hazards group InfraRed Precipitation with Stations (CHIRPS) rainfall showed the least bias and error (ME ≈ −0.2–0.2 mm, MAE ≈ 0.5–2 mm), and the best agreement (COR ≈ 0.8), with station rainfall data. In contrast FLDAS had the higher bias and error and the lowest agreement and Global Precipitation Mission/Tropical Rainfall Measurement Mission (GPM/TRMM) data were intermediate. This information can inform the selection of geospatial data products for use in climate and disease research and applications.

## 1. Introduction

Vector-borne diseases remain a major public health concern in developing countries. In Ethiopia, more than 75% of the area (elevation < 2000 m asl) of the country is considered to be malarious or potentially malarious, and 68% of the population (>50 million people) live in these malaria-impacted areas [1,2]. Malaria transmission in Ethiopia is seasonal and depends on favorable climatic and ecological factors for the growth of mosquito populations and the transmission of malaria parasites. In the Amhara Region, malaria cases typically peak at the end of the rainy season between September and December, and in some areas also have a smaller peak at the beginning of the rainy season in May and June [3,4]. Understanding how environmental factors trigger these seasonal outbreaks provides the basis for malaria early warning systems that can forecast changes in malaria transmission risk based on climate variability [5,6,7]. To achieve this goal, it is also essential to have reliable data streams for monitoring variations in climate. This study explores the suitability of several satellite remote-sensing and gridded meteorological datasets for measuring daily temperature and precipitation in the Ethiopian highlands.

Temperature and precipitation are fundamental environmental variables that influence spatial and temporal patterns of malaria risk. Temperature drives mosquito population dynamics and malaria transmission by affecting multiple vital rates, including mosquito egg production, growth, and survival as well as infection probabilities and the rate of parasite development within the mosquito vector [8]. As a result, the basic reproductive rate (R_0_) of malaria is highest at an optimal temperature and decreases at higher and lower temperatures. In the East African highlands, cool temperatures at high elevations historically limited malaria transmission to elevations below approximately 2000 m [9]. More recently, there is evidence that temperatures have increased in higher-elevation regions of Ethiopia [10] and that these higher temperatures have resulted in increased malaria cases in the highlands [11]. Precipitation is the ultimate source of water for larval habitats, and therefore often has a lagged relationship with malaria transmission in drier environments where water for breeding habitats is a limiting factor [3,6,12]. However, the effects of rainfall on the development of pools suitable for mosquito breeding are strongly conditioned by hydrological factors such as terrain and soils [13]. Also, very heavy or continuous rains can wash out breeding habitats and create large water bodies and rapid flows that are not suitable for the larvae of malaria vectors [14]. 

Research on climate–malaria relationships in Ethiopia is important to further our understanding of the complex linkages between climate variation and disease transmission, and this knowledge can be applied to predict the locations of malaria risk hotspots and the timing of malaria outbreaks. Many of these studies have relied on in situ data collected at ground-based weather stations [12,15,16], which are generally considered the gold standard for meteorological data [17]. However, ground-based data are sparely distributed in space, particularly in Africa, and often have data gaps in their time-series records. [18]. Therefore, other research studies and applications have used gridded environmental data collected by Earth-observing satellites. Many of these datasets encompass the entire globe and are updated daily, making them suitable for monitoring the environmental factors associated with malaria risk. The satellite remote-sensing data that have been used for malaria research include precipitation estimates [19], land-surface temperature (LST) [20], the normalized difference vegetation index (NDVI) and other spectral indices derived from optical-infrared remote sensing [21]. Gridded meteorological datasets, which are developed using various combinations of interpolated meteorological data, satellite observations, and models, provide another source of spatially continuous environmental data that has been used to assess climate-disease relationships [22].

The main objective of this study was to explore the accuracy of several types of environmental data derived from satellite remote-sensing and gridded climate data products for measuring temperature and precipitation in the Amhara Region of Ethiopia. This research was motivated by our ongoing work on the Epidemic Prognosis Incorporating Disease and Environmental Monitoring for Integrated Assessment (EPIDEMIA) project, which aims to develop an early-warning system for epidemic human malaria in Ethiopia. The early warning system includes software that integrates temperature, precipitation, and vegetation data from Earth-observing satellites to monitor the environmental risk factors for malaria outbreaks [23,24]. Forecasts are made with empirical time series models that use distributed lags to capture the delayed effects of environmental fluctuations on malaria cases [25]. To make effective forecasts, it is essential that the environmental data used to drive the models is as accurate as possible, and that major sources of error and systematic biases are well understood. 

There has been considerable interest in the use of satellite rainfall products for monitoring crop productivity and flooding in Ethiopia. Several previous studies have compared multiple satellite rainfall products with meteorological station data, but these comparisons were made at seasonal or monthly rather than daily time scales [18,26]. Another study assessed the relationships between remotely sensed land-surface temperature (LST) measurements and daily air temperature observations from meteorological stations but did not explore other potential sources of geospatial temperature data [20]. To expand our understanding of remotely sensed climate indices and gridded climate data products in the Ethiopian highlands, we compared multiple spatial precipitation and temperature data products over a 13-year period. with observations from twenty-two meteorological stations in the Amhara Region of Ethiopia. To ensure the results are relevant to the EPIDEMIA project, we selected a subset of the wide range of available data sources that are the most suitable for malaria early warning. These datasets are all have a daily temporal resolution and are available as continuous gridded datasets with latency ranging from a few days to approximately one month. Our main objectives were to: (1) compare the bias, error, and correlation of daily rainfall estimates between spatial climate datasets and meterological station observations; and (2) assess the geographic distribution of these accuracy metrics across elevation gradients within the study area.

## 2. Study Area, Data and Methods 

### 2.1. Study Area

**Figure 1:**
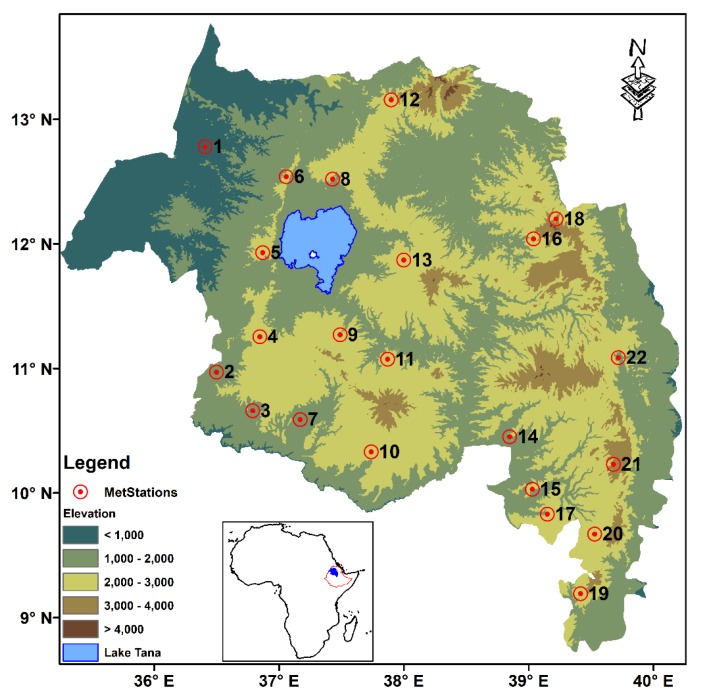
Location map of the study area with meteorological stations superimposed over an elevation map of the Amhara Region, Ethiopia. Names and descriptions for these stations are given in Table 1.

The study area encompassed 22 meteorological stations distributed throughout the Amhara Region of Ethiopia (Figure 1). Elevation in the region varies from 500 m at the northwestern border with Sudan to more than 4000 m mainly in the Semen (Northern) Mountain Ranges (Table 1). The highest peak in the Semen Mountains peaks is Ras Dejen, which has a height of 4620 m. Mean annual rainfall, which varies from 500 mm to 2000 mm, is highest in the southwestern part of the region, and generally decreases to the east. Rainfall is highly seasonal, with the heaviest rains falling from June through September, and dry conditions occurring from October through February. Average annual air temperature ranges from 27 °C at the lowest elevations to 16 °C in the highlands.

### 2.2. Data

Daily meteorological data from 22 stations were obtained for the period 2003–2016. Measurements included minimum and maximum daily temperatures and total daily precipitation. Minimum and maximum daily temperature were summarized to calculate mean daily temperature. These data were obtained via a data-sharing agreement with the Ethiopian National Meteorological Agency.

Publicly available geospatial data were obtained from five different sources and included three temperature datasets and three precipitation datasets (Table 2). Land-surface temperature, including daytime and night-time observations, were MYD11A1 daily land-surface temperature (LST) from the MODerate-resolution Imaging Spectroradiometer (MODIS) on board the National Aeronautics and Space Administration (NASA) Aqua spacecraft. Air temperature estimates were obtained from the Global Land Parameter Data Record (LPDR) which is derived using passive microwave data from Advanced Microwave Scanning Radiometer on Earth Observing Satellite (AMSR-E) on board the NASA Aqua MODIS satellite (May 2002–November 2011) and Advanced Microwave Scanning Radiometer 2 (AMSR2) on board the Japan Aerospace Exploration Agency (JAXA) Global Change Observation Mission 1st Water (GCOM-1W) satellite (July 2012–present; [27]). These sensors have a similar ascending and descending paths of 1:30 PM and AM respectively. The temporal data gap between AMSR-E and AMSR2 was filled by data from the Microwave Radiation Imager (MWRI) on board the Chinese FengYun 3B (FY3B) satellite [27]. This blended AMSR-E and AMSR2 data will be referred hereafter as AMSR.

Precipitation data were obtained from the Global Precipitation Mission (GPM) Integrated Multi-Satellite Retrievals for GPM (IMERG) dataset version 6. This multisatellite merged passive and active microwave dataset combines newer data from the GPM satellite mission with older data from the Tropical Rainfall Measurement Mission (TRMM), and had a spatial resolution of 0.1 degree. The Climate Hazards Group InfraRed Precipitation with Stations (CHIRPS) dataset combines satellite precipitation estimates with in situ station rainfall data and has a spatial resolution of 0.05 degree [28]. 

Data from the Famine Early Warning Systems Network (FEWS-Net) Land Data Assimilation System (FLDAS) included gridded meteorological forcing fields and modeled hydrological variables [29]. The FLDAS air temperature field is derived from the National Oceanic and Atmospheric Administration (NOAA) Global Data Assimilation System (GDAS) and NASA Modern Era Reanalysis for Research and Applications version 2 (MERRA-2) datasets. The FLDAS precipitation field integrates multiple sources of data, including the African Rainfall Estimation version 2.0 (RFE2), CHIRPS, and GDAS. We used the National Centers for Environmental Prediction/Oregon State University/ Air Force/Hydrologic Research Lab (NOAH) model derived 0.1° × 0.1° daily Eastern Africa Region data product. 

### 2.3. Methods 

**Figure 2:**
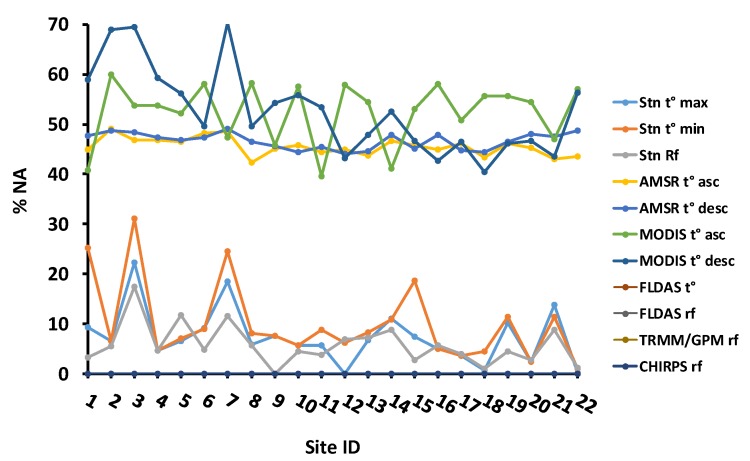
Percentage of daily missing data (% NA) of temperature and rainfall for 2003–2016. Note that Site ID in this figure corresponds to station labels in Figure 1 and Table 2. Note also that station, AMSR, and MODIS temperature are measured daytime and nighttime while the rest of the variables are daily. Note that the % NA values are zero at all sites for FLDAS, Tropical Rainfall Measurement Mission/Global Precipitation Mission (TRMM/GPM), and CHIRPS.

We used 14 years (2003–2016) of meteorological station and geospatial data. Pixel level daily time series data at their native spatial resolution (Table 2) were extracted for pixels that overlapped with the meteorological stations. Figure 2 presents the proportions of missing data from the original time series for both the station and satellite/interpolated datasets.

We calculated mean error (ME), mean absolute error (MAE), and correlation (r) statistics to evaluate the accuracy of satellite/interpolated datasets in relation to station data. ME estimates the average error and helps to capture the bias of the satellite/interpolated data in relation to the observed in situ data. A positive value indicates an overestimate of the satellite/interpolated data, whereas a negative value indicates an underestimate of the satellite/interpolated data as compared to the observed in situ data. ME values of zero are the perfect score (Table 3). The MAE of a satellite/interpolated dataset with respect to in situ data is the mean of the absolute values of the individual prediction errors on over all instances in the satellite/interpolated data. Each prediction error is the difference between the in situ data value and the satellite/interpolated data value for the instance. The Pearson correlation coefficient (r) is used to measure the goodness of fit and linear association between two variables. It measures how well the satellite/interpolated data corresponds to the observed in situ data. It helps to capture concordance in day-to-day variability of the satellite/interpolated data with in situ data. Its value ranges between −1 to 1, in which one indicates the perfect score.

Before conducting any further analyses, we applied a 7-day retrospective moving average smoothing on the study time series data variable to fill gaps that occur due to satellite swath width, cloud cover, aerosol, dust and other factors [30,31,32]. For the AMSR and MODIS twice-daily data, the daytime and nighttime values were averaged to get one daily value per 24 h.

**Figure 3:**
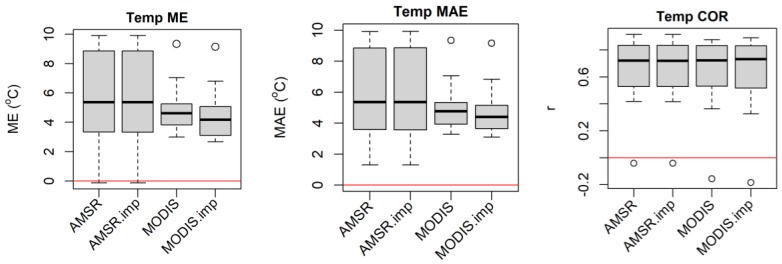
Comparative accuracy statistics box plot of non-imputed and imputed daily AMSR air temperature and MODIS LST for 22 meteorological stations distributed in the Amhara Region, Ethiopia for 2003–2016. Note the distinct differences of comparative box plots for the MODIS data mean error (ME) and mean absolute error (MAE).

The MODIS LST data has many missing values during the rainy and cloudy season in this part of the world. To decide whether to use interpolation methods to impute the remaining mission values that are available in the MODIS and AMSR average daily variables that were not filled by the retrospective moving average smoothing procedure, we compared the non-imputed and imputed datasets. The imputed time series data (particularly the MODIS dataset) showed smaller ME, MAE, and better r with the in situ time-series data (Figure 3). Thus, we decided to impute the missing values in the MODIS and AMSR satellite datasets for our study. The advantage of the imputation is not only improving the quality of the data, but most importantly, there will be available data for every time period of the data (daily in our case), so as to facilitate basic research as well as operational applications.

We tested two methods of missing value imputation: linear interpolation and seasonally decomposed missing value imputation (SDMVI) with the interpolation algorithm [33,34]. The SDMVI method with the interpolation algorithm first removes the seasonal component of the time-series data and then performs the imputation on the de-seasonalized data using linear interpolation or carrying the last observed value forward. Then, the seasonal component of the time series is added back to the imputed data [34]. For data that have strong seasonality, the SDMVI and the Kalman Smoother have been found to produce the best results [33,34]. In our case, we used the SDMVI method in our preliminary analyses found that this method is computationally very efficient and has similar accuracy with the Kalman Smoother for selected sample sites. 

**Figure 4:**
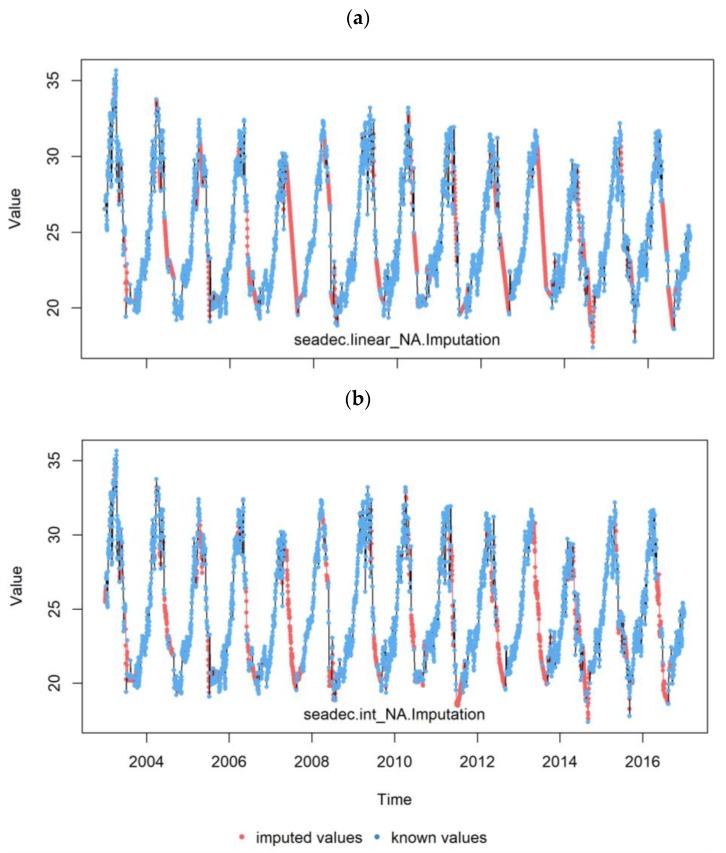
MODIS LST missing data imputation using (**a**) simple linear and (**b**) seasonal decomposition with interpolation algorism methods for Lay Birr meteorological station for 2003–2016.

**Figure 5:**
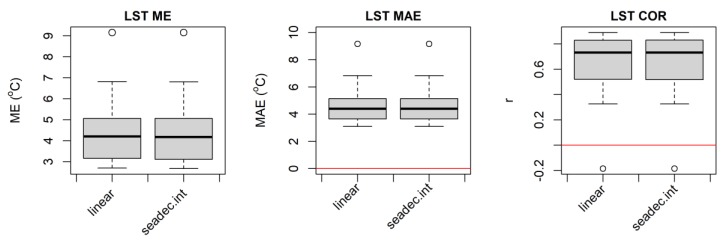
Daily MODIS LST comparative accuracy statistics box plots from two missing data imputation methods (linear and seasonal decomposition with interpolation algorithm) for 22 meteorological stations in the Amhara Region, Ethiopia for 2003–2016.

We compared accuracy statistics values of MODIS LST versus station air temperature using the two methods of missing value imputation (Figure 4). All the accuracy statistics results (ME, MAE, and r) from the SDMVI method showed slight improvements compared to those from the simple linear interpolation method (Figure 4 and Figure 5). Thus, we used SDMVI for the MODIS and AMSR satellite data missing value imputation.

We generated binned scatterplots of satellite/interpolated datasets as a function of corresponding station datasets. Hexagon binning is a form of bivariate histogram that is useful for visualizing the structure in datasets with large numbers of observations. The number of points falling in each tessellated regular grid of hexagons are counted and stored in a data structure [35,36,37,38].

We analyzed temperature and rainfall records by season. In Ethiopia, there are three seasons: the main rainy season (locally called Kiremt; June–September), the dry season (Bega; October–February), and the small rainy season (Belg; March–May) [31]. In our study area, Amhara Region, the rainfall during the small rainy season is relatively low. Therefore, we merged the small rainy season into the dry season (Dry season: October–May). Then, we generated accuracy assessment statistics for both the rainy and dry season, to evaluate influence of seasonality on the accuracy of the satellite environmental data record in the study area.

## 3. Results and Discussion

### 3.1. Temperature and Rainfall Time Series

**Figure 6:**
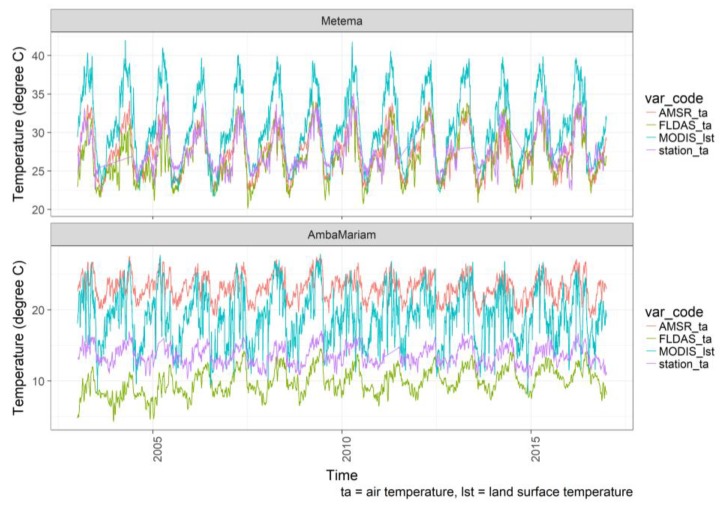
Time series plot of temperature from remote sensing satellite (AMSR air temperature (ta) and MODIS LST), interpolated (FLDAS ta), and meteorological station (station ta) datasets at a lowland (Metema), and highland (Amba Mariam) stations, Northwest Ethiopia, for 2003–2016. Note the y-axis scale difference.

All satellite/interpolated temperature datasets tracked the temperature seasonality observed in the meteorological station data (Figure 6). In general, the satellite/interpolated temperature data showed a better seasonality and magnitude agreement with station temperature data in the lowlands (Figure 6 Metema) than in the highlands (Figure 6 Amba Mariam). AMSR and FLDAS air-temperature data displayed a close agreement, while MODIS land-surface temperature showed overestimation in the lowlands. In the highlands, AMSR and MODIS overestimated station temperature compared to the station data, while FLDAS underestimated station temperature. MODIS LST has larger seasonal variation compared to AMSR, FLDAS, and station temperature at both lowland and highland sites. 

Note that land-surface temperature and near surface air temperature measure different aspects of the environment. Thus LST, a measure of the temperature of the land surface is expected to differ from station measurements of station temperature at 2 m above the Earth’s surface. However, LST is often used as a proxy for air temperature. Vancutsem et al. [20] estimated air temperature using MODIS LST over Africa with MAE of 1.73 °C and a standard deviation of 2.4 °C for the nighttime temperature, while estimation of daytime temperature strongly varied with seasonality, ecosystem, solar radiation, and cloud-cover. Further discussion of seasonal differences between land-surface and air temperature is provided in Section 3.3. 

**Figure 7:**
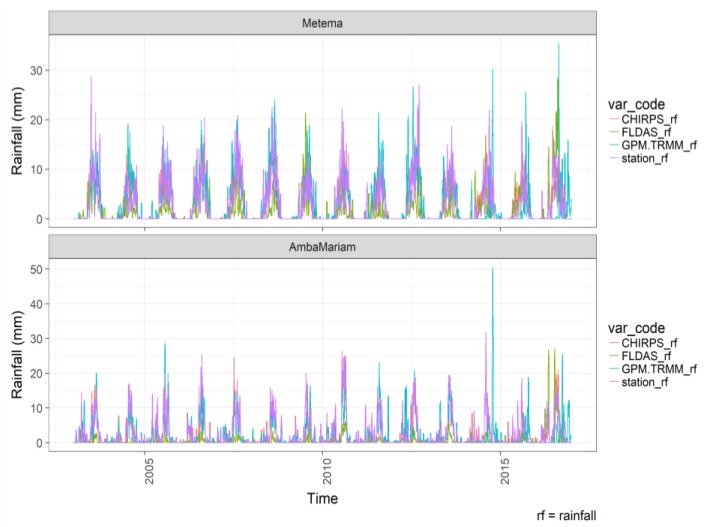
Time series plot of rainfall from remote sensing satellite (GPM.TRMM_rf), model driven (CHIRPS_rf, FLDAS_rf), and meteorological station (stations_rf) datasets at a lowland (Metema), and highland (Amba Mariam) stations, Northwest Ethiopia, for 2003–2016. Note the y-axis scale difference.

The CHIRPS and TRMM rainfall data in Figure 7 showed similar magnitude and seasonality with the station rainfall datasets for most of the analyzed meteorological stations (Figure 2). FLDAS rainfall underestimates station rainfall, while CHIRPS followed by TRMM-GPM showed a good agreement.

### 3.2. Station and Satellite/Reanalysis Datasets’ Binned Scatterplots

**Figure 8:**
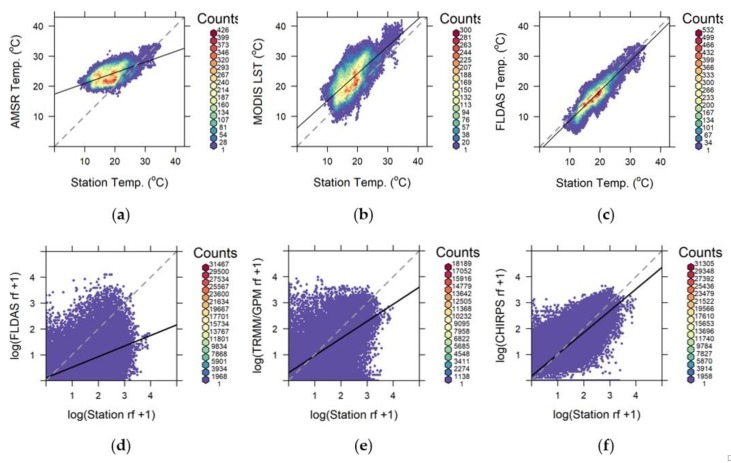
Binned scatter plots of temperature (**a**–**c**) and rainfall (**d**–**f**) from satellite/interpolated and station datasets. The light gray diagonal dashed lines are 1:1 lines, while the solid-black lines are fitted linear regression lines. Note the color variation of the hexagons. Each hexagon holds one or more number of data points, with red-highest and purple-lowest number of points. Note also the convergence/divergence of the linear regression line from the 1:1 diagonal line.

The binned scatter plots in Figure 8 showed that FLDAS temperature was strongly correlated with station temperature, with only slight underestimation (Figure 8c). However, AMSR temperature displayed a strong overestimation bias at lower temperature, which are typically at higher elevations (Figure 8a). MODIS also had consistent overestimation bias (Figure 8b), but it was closer to the station data than AMSR temperature. The rainfall-binned plots were dominated by zero and lower values in all the measurements (Figure 8d–f). The regression line for the CHIRPS rainfall was close to the 1:1 diagonal line of the binned plot (Figure 8f), while that of FLDAS displayed the largest underestimation bias (Figure 8d).

### 3.3. Accuracy Statistics 

#### 3.3.1. Box Plots

**Figure 9:**
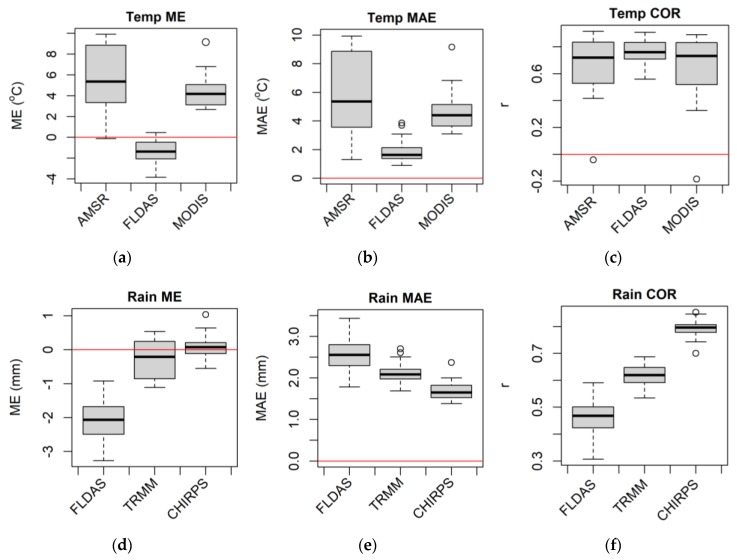
Pixel-level satellite/interpolated temperature (**a**–**c**) and rainfall (**d**–**f**) daily data accuracy statistics box plots over 22 meteorological stations distributed in Amhara Region, Ethiopia for 2003–2016.

The FLDAS temperature showed the lowest bias (ME ≈ 1–3 °C; Figure 9a), and error (MAE ≈ 1–3 °C; Figure 9b) and the strongest correlation with the day-to-day variability of station temperature (COR ≈ 0.7–0.8; Figure 9c). In contrast, AMSR temperature showed a larger bias (ME ≈ 3–9 °C; Figure 9a), and error (MAE ≈ 3–9 °C; Figure 9b), and lower agreement (COR ≈ 0.5–0.8; Figure 9c). CHIRPS rainfall showed the least bias and error (ME ≈ −0.2–0.2 mm, MAE ≈ 0.5–2 mm; Figure 9d and e respectively), and the best agreement (COR ≈ 0.8; Figure 9f), with the station rainfall data. FLDAS rainfall displayed larger bias and error (ME ≈ −1.5–−2.5 mm, MAE ≈ 1.5–2 mm; Figure 9d and e respectively), and lower agreement (COR ≈ 0.4–0.5; Figure 9f).

The lower errors in the CHIRPS data are expected, as this dataset is blended with pentadal and monthly station datasets [28]. Previous studies found that the accuracy of CHIRPS rainfall products is significantly better compared to other satellite/interpolated rainfall products [39,40,41]. Duan et al. [40] evaluated eight gridded precipitation products in Adige Basin, Italy with gridded rain gauge data at 0.25 spatial and daily, monthly and annual temporal resolution. The satellite products that they used include TRMM 3B42, raw Climate Prediction Center’s morphing technique (CMORPH_RAW), bias-corrected CMORPH (CMORPH_CRT), satellite-gauge blended (CMORPH_BLD), Precipitation Climate Data Record (PCDR), Princeton Global Forcings (PGF), CHIRPS, and Global Satellite Mapping of Precipitation project Moving Vector with Kalman-filter product (GSMaP_MVK). Overall statistical metrics revealed that the CHIRPS, TRMM and CMORPH_BLD ranked as the top three best performing products, while the PGF performed worst. Bayissa et.al., evaluated five satellite and interpolated rainfall products for the Upper Blue Nile Basin, Ethiopia ([41]; CHIRPS, Precipitation Estimation from Remotely Sensed Information using Artificial Neural Networks (PERSIANN), Tropical Applications of Meteorology using SATellite and ground-based observations (TAMSAT) African Rainfall Climatology And Time series (TARCAT), TRMM, and Africa Rainfall Estimate Climatology version 2 (ARC) 2.0). CHIRPS performed best of all the products with lower bias (close to one) and mean error at decadal, monthly and seasonal time scales. Among six rainfall products including TMPA, CMORPH, PERSIANN, European Center for Medium range Weather Forecast (ECMWF), ECMWF Re-Analysis, (ERA) Interim Reanalysis, and Multi-Source Weighted-Ensemble Precipitation (MSWEP), adjusted CMORPH exhibited the best accuracy of the wet season rainfall estimate, while MSWEP outperform both unadjusted and gauge adjusted ERA-Interim estimates over the upper Blue Nile basin, Ethiopia [42]. The microwave based products TRMM (TMPA 3B42RT) and CMORPH outperformed the infrared-based product PERSIANN over Ethiopian river basins [43]. PERSIANN tended to underestimate rainfall by 43%, while CMORPH tends to underestimate by 11% and TMPA 3B42RT tends to overestimate by 5% [43]. A study that evaluated the MSWEP rainfall reanalysis product over Africa found that it had no obvious advantages compared to Global Precipitation Climatology Centre (GPCC), CHIRPS or Agricultural Climate Forecast System Reanalysis (AgCFSR) [44]. In particular, MSWEP was unable to capture major hydro-climate extremes over west, east and southern Africa, where it underestimated compared to CHIRPS [44].

#### 3.3.2. Spatial Distribution of Accuracy Statistics

**Figure 10:**
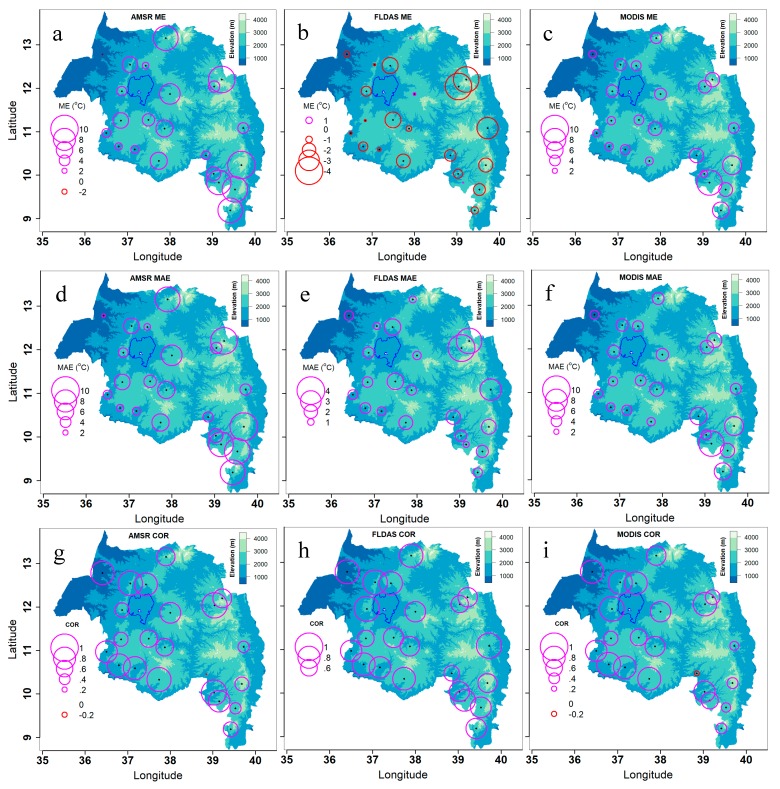
Pixel-level satellite/interpolated daily temperature data accuracy statistics spatial distribution for 22 meteorological stations in Amhara Region, Ethiopia for 2003–2016. Note the legend-scale differences.

**Figure 11:**
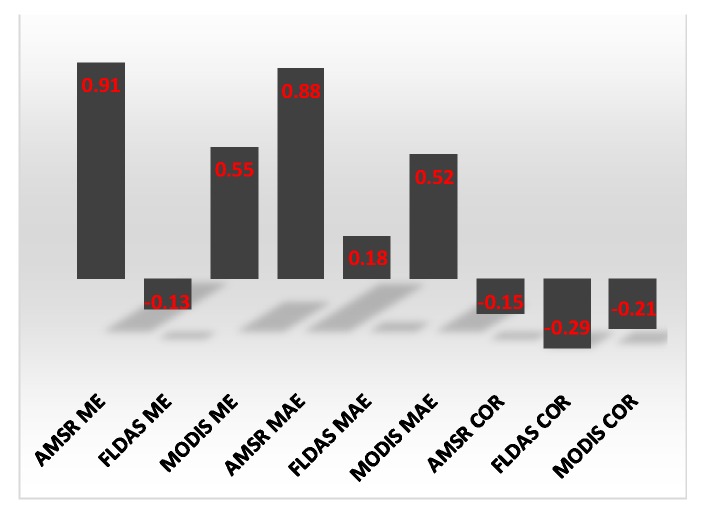
Bar graph of the correlations between elevation and accuracy statistics for the various satellite/interpolated data products.

**Figure 12:**
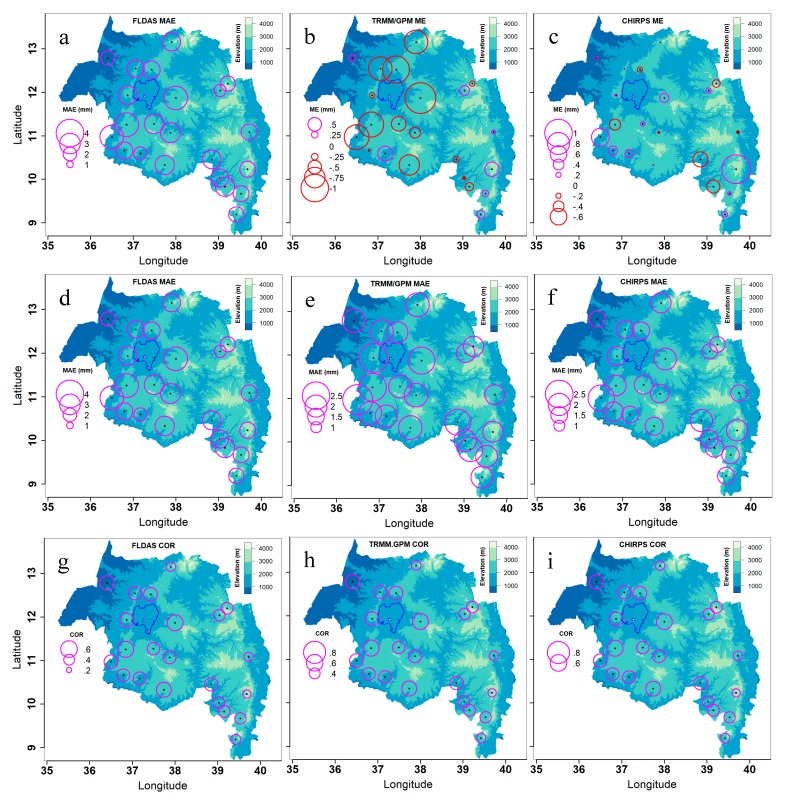
Pixel-level satellite/interpolated daily rainfall data accuracy statistics spatial distribution for 22 meteorological stations in Amhara Region, Ethiopia for 2003–2016. Note the legend scale differences.

AMSR and MODIS temperature displayed a significant difference in bias and error between lowlands and highlands (Figure 10a,c,d,f). AMSR ME and MAE were positively correlated with elevation with r of 0.91 and 0.88 respectively (Figure 10a,d; Figure 11), while the correlations of MODIS ME and MAE were 0.55 and 0.52, respectively (Figure 10c,f; Figure 12a). FLDAS temperature ME and MAE did not have strong correlations with elevation (Figure 10b,e; Figure 11). Generally, the accuracy of all satellite/interpolated temperature products decreased with elevation, and this association was stronger for FLDAS than MODIS and AMSR (Figure 10g–i; Figure 11).

**Figure 13:**
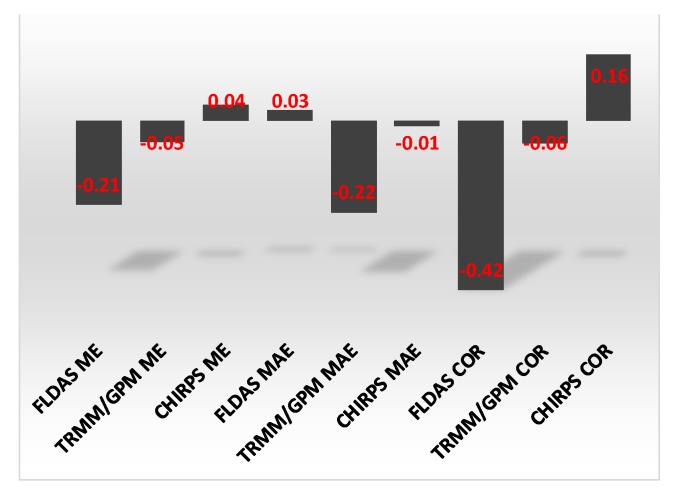
Bar graph of the correlations between elevation and accuracy statistics for the various satellite/interpolated precipitation products.

FLDAS rainfall bias decreased with elevation (Figure 12a). The correlation between the bias and elevation was −0.21 (Figure 13). The MAE for TRMM/GPM was also inversely correlated with elevation (r = −0.22). The correlation between FLDAS and station rainfall significantly decreased with elevation with an r of −0.42 (Figure 12g; Figure 13), while that of CHIRPS and station rainfall increased with elevation (Figure 12g; Figure 13).

#### 3.3.3. Accuracy Statistics Seasonal Distributions

**Figure 14:**
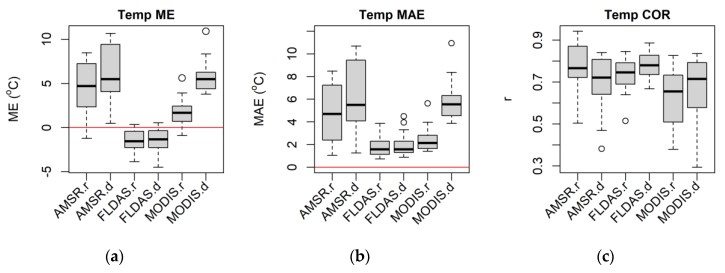

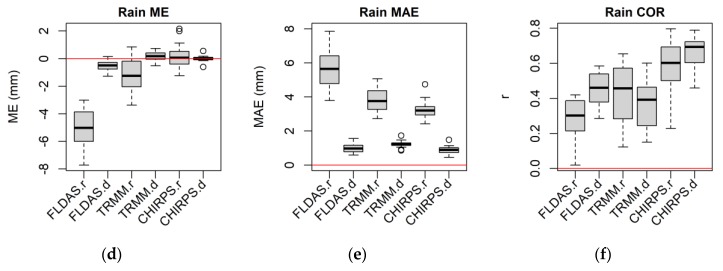
Pixel-level satellite/interpolated temperature (**a**–**c**) and rainfall (**d**–**f**) seasonal data (seasonal averages from daily data) accuracy statistics box plots over 22 meteorological stations distributed in Amhara Region, Ethiopia for 2003–2016. The “.r” is for rainy and “.d” is for dry seasons.

Both ME and MAE of AMSR and MODIS temperature were larger during the dry season than the rainy season (Figure 14a,b). Dry season MODIS and FLDAS temperature data correlation with corresponding station data were higher in the rainy season compared to the dry season, while AMSR data showed the reverse (Figure 14c). Land-surface temperature and near surface air temperature measure different aspects of the environment [45]. Seasonality of similarities and differences of these two temperatures depends on the seasonal dynamics of the Bowen ratio [46]. The Bowen ratio is the ratio of the sensible heat flux to latent heat flux. During the rainy season, much of the sensible heat flux is consumed for evapotranspiration, which in turn increases the latent heat flux. There is a lower Bowen ratio during rainy season, as sensible heat flux become lower and latent heat flux become higher [30,32,45,46]. LST and air temperature tend to equilibrate in the rainy season due to low sensible heat flux.

In the dry season, all FLDAS, TRMM/GPM, and CHIRPS rainfall data ME and MAE values were lower compared to those of the rainy season records (Figure 14d,e). Dry season FLDAS and CHIRPS rainfall data showed higher correlation with corresponding station rainfall data compared to the rainy season records, while rainy season TRMM/GPM rainfall showed higher correlation than the dry season data (Figure 14f). The station and remotely sensed rainfall data all had lower values during the dry season, which could contribute to the lower error values than in the rainy season when rainfall amounts are high and there is more potential for satellite and interpolated measurements to deviate from the station measurements.

## 4. Conclusions and Recommendations

Vector-borne diseases, such as malaria, are still major public health concerns in developing countries, such as Ethiopia. Forecasting and early warning of such diseases is hindered by scarce and poor-quality meteorological station datasets in the region. This study was aimed at evaluating the accuracy of satellite-based environmental datasets with meteorological station datasets. We found that FLDAS temperature binned scatter plots were closely associated with station temperature, while AMSR temperature displayed an overestimation bias at lower temperature in highland areas. MODIS LST had a consistent overestimation bias, but was more accurate than AMSR temperature. The regression line for the CHIRPS rainfall is close to the 1:1 diagonal line of the binned plot, while that of FLDAS rainfall displayed the largest underestimation bias. The FLDAS interpolated temperature data showed the lowest bias (ME), and error (MAE), and best agreement (COR) with corresponding station temperature data. In contrast, AMSR temperature showed the largest bias and error and weakest correlations. CHIRPS rainfall showed the least bias and error, and best agreement with station rainfall data. FLDAS rainfall displayed the largest bias and error, and weakest correlations.

The FLDAS air temperature and CHIRPS rainfall datasets can provide sources of meteorological data that are strongly associated with daily patterns of station temperature and rainfall data within the study area and potentially in other areas throughout in the world. However, the FLDAS daily products are no longer being produced and FLDAS now provides only a global, monthly product with latency of greater than one month. Similarly, the CHIRPS rainfall dataset has a latency of longer than a month, which limits its utility for early-warning applications. Thus, MODIS LST and AMSR temperature products may be useful in many situations because they have relatively strong day-to-day correlations with station temperatures despite their higher ME and MAE. The TRMM/GPM IMERG daily rainfall data can also provide rainfall estimates with low bias that are only slightly less accurate than CHIRPS and are superior to the FLDAS rainfall product. We also suggest that the development of new, regionally calibrated gridded meteorological datasets that combine satellite observations and stations measurements, such as those developed by the Enhancing National Climate Services (ENACTS) project [47], will be an important step toward providing better data to support climate and malaria research and applications. Overall, more research will be needed to better understand the strengths and limitations of various sources of meteorological data and to determine how the underlying differences influence climate and health assessments.

## Figures and Tables

**Table 1:** List of meteorological stations mapped in Figure 1 with their geographic locations and elevation. Stations are sorted by longitude. Note that the station number (SN) in this table corresponds to station labels in Figure 1.

SN	Station Name	Long	Latitude	Elevation
1	Metema	36.41	12.78	790
2	Chagni	36.50	10.97	1614
3	Ayehu	36.79	10.66	1771
4	Dangila	36.85	11.25	2116
5	Shahura	36.87	11.93	2205
6	Aykel	37.06	12.54	2254
7	Lay Birr (SF)	37.17	10.59	1707
8	Gondar A.P.	37.43	12.52	1973
9	Adet	37.49	11.27	2179
10	Debre Markos	37.74	10.33	2446
11	Motta	37.87	11.07	2417
12	Debark	37.90	13.16	2836
13	Debre Tabor	38.00	11.87	2612
14	Majete	38.85	10.45	2000
15	Alem Ketema	39.03	10.03	2280
16	Lalibela	39.04	12.04	2487
17	Enewari	39.15	9.83	2561
18	Amba mariam	39.22	12.2	2990
19	Shola Gebeya	39.42	9.19	2500
20	Debre Berhan	39.53	9.67	2750
21	Mehal Meda (RS)	39.68	10.23	3084
22	Combolcha	39.72	11.09	1857

**Table 2:** Satellite interpolated and meteorological datasets used in this study with their characteristics and descriptions.

SN.	Dataset		Version	Spatial Resolution	Temporal Resolution
	Data	Data Variables			
1	AMSR ¹	Air temperature (*ta*; ~2 m height)	2.0	25 km	Daily (max and min)
2	FLDAS ²	Air Temperature (ta)	-	10 km	Daily
		Rainfall (rf)	-	10 km	Daily
3	CHIRPS ³	Rainfall	2.0	5km	Daily
4	MODIS ⁴	MYD11A1 (Land-Surface Temperature—LST)	Collection 6	1 km	Daily (max and min)
5	GPM ⁵	Rainfall	06	10 km	Daily
6	Met station	Temperature	-	-	Daily (max and min)
		Rainfall	-	-	Daily

¹ http://files.ntsg.umt.edu/data/LPDR_v2/; ² https://ldas.gsfc.nasa.gov/FLDAS/FLDASmodel.php; ³ http://chg.geog.ucsb.edu/data/chirps/; ⁴ https://cmr.earthdata.nasa.gov/search/concepts/C203669661-LPDAAC_ECS.html; ⁵ https://disc.gsfc.nasa.gov/datasets/GPM_3IMERGDF_V06/summary.

**Table 3:** Description of the statistical metrics used in the evaluation of environmental data products.

Statistical Metric	Equation	Range	Unit	Optimal Value
Mean error (ME)	$\frac{1}{n}\overset{n}{\underset{i=1}{\sum }}\left(y_{i}-x_{i}\right)$	−∞ to ∞	°C or mm	0
Mean absolute error (MAE)	$\frac{1}{n}\overset{n}{\underset{i=1}{\sum }}\left|y_{i}-x_{i}\right|$	0 to ∞	°C or mm	0
Correlation coefficient (r)	$\frac{\sum_{i=1}^{n}(x_{i}-\bar{x})\left(y_{i}-\bar{y}\right)}{\sqrt{\sum_{i=1}^{n}\left(x_{i}-\bar{x}\right)²}\sqrt{(\sum_{i=1}^{n}\left(y_{i}-\bar{y}\right)²}}$	−1 to 1	None	1

*n* is sample size; *x_i_* are the observed measurements from the meterological stations, *y_i_* are the predicted values from remotely sensed and interpolated data products, *i* indexes the measurements by time and location; and $\bar{x}$ and $\bar{y}$ are the mean values of the observations and predictions.
